# Polymer-Coated Iron Oxide Nanoparticles as an Effective Tool for Histamine Extraction: Synthesis, Characterization, and Application

**DOI:** 10.1021/acsomega.5c08198

**Published:** 2025-10-10

**Authors:** Marco Reindl, Anjali Karn, Verena Zach, Sebastian P. Schwaminger

**Affiliations:** † NanoLab, Division of Medicinal Chemistry, Otto Loewi Research Center, 31475 Medical University of Graz, Neue Stiftingtalstraße 6, 8010 Graz, Austria; ‡ BioTechMed-Graz, Mozartgasse 12, 8010 Graz, Austria

## Abstract

Histamine accumulation in food products poses significant health risks, necessitating efficient removal strategies to ensure food safety and quality. This study presents the synthesis, characterization, and optimization of poly­(acrylic acid-*co*-methacrylic acid)-coated iron oxide nanoparticles [ION@P­(AA-*co*-MAA)] as adsorbents for histamine extraction from food-mimicking buffers. Using a combined Plackett–Burman and central composite design approach, we identified the monomer-to-iron oxide nanoparticle (IONP) ratio and polymerization temperature as critical synthesis parameters significantly influencing nanoparticle size (hydrodynamic diameter ranging from 182 to 801 nm), surface charge (ζ potential from −39.1 to −23.8 mV), polymer coating thickness (weight loss between 3.3% and 5.9%), and histamine adsorption capacity (77.3 to 130.5 mg/g under optimized conditions). The optimized ION@P­(AA-*co*-MAA) particles demonstrated high adsorption capacities, up to 55 mg/g in soy sauce-mimicking buffer, with efficient magnetic separation facilitated by their superparamagnetic properties. Adsorption isotherm analysis revealed heterogeneous binding sites consistent with polymer coating characteristics, while reusability tests confirmed practical potential in salt-rich environments. This work advances the development of functionalized magnetic nanoparticles for histamine removal, offering a scalable, reusable platform to enhance food safety and support low-histamine food production.

## Graphical abstract

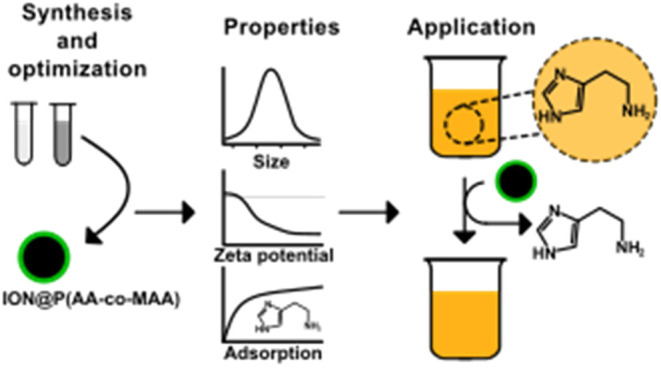

## Introduction

Histamine is a biogenic amine (BA) formed mainly through microbial decarboxylation of histidine^1^ and commonly found in fermented foods such as cheese, sausage, vegetables, wine, and fish.^2,3^ While small amounts are detoxified by intestinal amine oxidases,^4,5^ excessive intake or impaired enzyme function can cause adverse effects, particularly in individuals with histamine intolerance (HIT).^6^

HIT is a nonimmunological disorder resulting from reduced histamine degradation, leading to symptoms like nausea, headaches, and respiratory issues.^7–9^ It affects approximately 1% of the population, with greater prevalence in middle-aged individuals.^10^ Contributing factors include genetic mutations in histamine-degrading enzymes, gut microbiota imbalances, and chronic illness.^7,8^ Diagnosis is clinical, based on symptom patterns and medical history, as no definitive biomarker exists.^7,11^ Management focuses on a low-histamine diet, DAO enzyme supplementation, and antihistamines.^7,8,11^

Current approaches for removing histamine from food include enzymatic degradation,^12^ microbial action,^13^ physical separation techniques like electrodialysis,^14^ and modified storage conditions^15^ (Table 1). While chromatographic and enzymatic assays remain central for detection and regulatory compliance, active removal strategies are gaining traction in both industrial and consumer settings.

**Table 1:** Summary of Histamine Extraction and Removal Techniques with Advantages of the Proposed Nanoparticle-Based Approach

**method**	**mechanism**	**limitations**	**ION@(AA-** *co* **-MAA) advantages**
enzymatic degradation	amine oxidase activity	substrate specificity, inhibition	stable, reusable adsorption platform
microbial action	bacterial degradation	strain-specific, inconsistent results	controlled adsorption
electrodialysis	electrochemical separation	high cost, complexity, partial removal	magnetic separation, simple handling
modified storage	inhibition of histamine-producing bacteria	does not remove existing histamine	active removal of histamine postformation
MISPE	molecular recognition	complex synthesis, costly	easier synthesis, high adsorption

Enzymes such as histamine dehydrogenase offer quick detection capabilities, though their performance can be limited by factors like substrate specificity and potential inhibition, which may impact both accuracy and sensitivity.^12^ Alternatively, microbial starter cultures from lactic acid bacteria and yeasts can be used to reduce histamine levels, as they produce amine-oxidase enzymes that break down histamine. Studies in cheese production have demonstrated that certain bacterial strains decrease histamine content by around 40–50% during the ripening.^13^

Electrodialysis has demonstrated up to 53.4% histamine reduction in fish sauce by optimizing current, pH, and flow rate,^14^ though its scalability is limited by cost and complexity. Other methods, such as high hydrostatic pressure, irradiation, or modified atmosphere packaging, inhibit histamine-producing bacteria but do not eliminate histamine already present.^15^ Similarly, molecularly imprinted solid-phase extraction (MISPE) allows for highly selective histamine removal at low detection limits (0.09 μg/L), but complex synthesis limits industrial adoption.^16^

Nanotechnology offers promising solutions for the removal of contaminants like histamine.^17^ In particular, iron oxide nanoparticles (IONPs) have gained significant interest because of their superparamagnetic behavior, chemical stability, and biocompatibility.^18^ These properties enable facile separation from complex food matrices using external magnetic fields, enhancing operational efficiency and reusability.^19^ However, bare IONPs tend to aggregate and have limited surface functionality for selective binding of different analytes.^20^ To overcome these limitations, the surface of IONPs can be functionalized with various materials, such as silica,^21^ lipids,^22^ or polymers,^23^ creating core–shell structures that enhance stability and binding behavior.

Among suitable coatings, poly­(acrylic acid-*co*-methacrylic acid) [P@(AA-*co*-MAA)] offers ionizable carboxylic groups for strong electrostatic binding to positively charged ligands like histamine. The added methyl group in MAA increases steric hindrance and hydrophobicity,^24^ while the copolymer structure ensures uniform carboxyl group distribution, enhancing ion accessibility and exchange capacity.^25,26^ This makes P­(AA-*co*-MAA) highly effective for capturing charged molecules via ionic and hydrogen bonding.

Importantly, polymers based on AA and MAA are EFSA-approved for food contact, confirming their safety for food-related applications.^27,28^ Cross-linked poly­(acrylic acid) sodium salts are approved as absorbent materials in food packaging, with no genotoxicity observed.^27^ Similarly, anionic methacrylate copolymers are permitted in solid food supplements, though no acceptable daily intake was established due to limited long-term toxicity data.^28^

In this study, we report the synthesis and optimization of poly­(AA-*co*-MAA)-coated IONPs for histamine adsorption. A Plackett–Burman design (PBD) was used to prescreen variables such as monomer concentration, polymerization time, and surfactant levels, followed by a central composite design (CCD) to refine synthesis parameters. Selected formulations were evaluated for cytotoxicity to ensure safe application, and histamine adsorption was assessed both in controlled systems and food-relevant matrices.

As summarized in Table 1, our approach offers several key advantages over existing techniques, including active removal of preformed histamine, high adsorption efficiency, ease of magnetic recovery, and potential for scalable production. This work introduces a practical, food-safe nanomaterial platform for targeted histamine removal, supporting food safety and dietary management in histamine-sensitive individuals.

## Results and Discussion

### Prescreening for Significant Factors

To identify the synthesis parameters that significantly influence the physicochemical properties of P­(AA-*co*-MAA)-coated IONPs [ION@P­(AA-*co*-MAA)], a PBD was employed (Table 2). This design is widely used for preliminary screening of multiple factors due to its efficiency in estimating main effects with a minimal number of experiments, as it focuses on estimating main effects without considering interactions.^29^ The primary aim was to distinguish between high- and low-impact variables affecting properties such as hydrodynamic diameter, ζ potential, and polymer thickness. The PBD enabled the identification of critical factors for further optimization more efficiently.

**Table 2:** Experimental Conditions Used for the PBD to Evaluate the Influence of Synthesis Parameters on Polymer-Coated IONP Formation^a^

condition	acrylic acid [μL]	methacrylic acid [μL]	SDS [mg]	APS [mg]	polymerization time [h]	temperature [°C]
1	824	1012	576.8	2191	4	60
2	51	63	2.9	274	0.5	60
3	824	1012	2.9	4381	0.5	80
4	51	63	2.9	137	0.5	60
5	51	63	576.8	137	4	80
6	824	1012	2.9	2191	4	60
7	824	1012	2.9	2191	4	80
8	51	63	576.8	274	4	60
9	51	63	576.8	137	0.5	80
10	51	63	2.9	274	0.5	80
11	824	1012	2.9	4381	4	60
12	824	1012	2.9	2191	0.5	60
13	51	63	576.8	274	4	80
14	824	1012	2.9	2191	0.5	80
15	824	1012	576.8	4381	0.5	80
16	51	63	2.9	137	4	80

a Each condition corresponds to a unique combination of high (+1) and low (−1) factor levels.

Five key factors were evaluated during prescreening: initiator-to-monomer ratio, monomer-to-IONP ratio, SDS concentration, polymerization time, and temperature (Table 2). These were selected for their known impact on polymer growth, nanoparticle functionalization, and colloidal stability.^30–32^ The initiator-to-monomer ratio influences radical availability, polymerization rate, and shell characteristics,^31,32^ affecting surface functionality critical for histamine binding. The monomer-to-IONP ratio determines coating thickness and uniformity,^30,32^ influencing binding site accessibility. SDS concentration affects dispersion and micelle formation,^31,32^ which in turn shape polymer morphology and particle stability. Polymerization time governs monomer conversion and network formation, balancing between incomplete coverage and excessive growth.^33^ Temperature affects polymerization kinetics and morphology by modulating radical activity.^33^ These parameters were assessed for their influence on ζ potential, hydrodynamic diameter, and polymer layer thickness to identify the most critical factors for optimizing the adsorption behavior of nanoparticles.

The successful coating of IONPs was confirmed by ATR-FTIR, dynamic light scattering (DLS), and transmission electron microscopy (TEM) (Figure 1 and Table 2), in comparison with bare IONPs (Figure S1A–E, Table S1). The hydrodynamic diameter (Z-average diameter) was determined by DLS and ranged from 105 to 233 nm (Figure 1A and Table 3). In comparison, bare IONPs showed a hydrodynamic diameter of 92 nm. These values align with previously reported hydrodynamic diameters for anionic polymer-coated IONPs.^23,34^ Ordinary least-squares multiple linear regression (OLS MLR) revealed that the ratio between monomers with a coefficient (β) of 24.3 (*p* = 0.004) and IONPs as well as the temperature (β = −15.8, *p* = 0.022) affected the hydrodynamic diameter significantly (Table S2), suggesting that more monomers in comparison to IONPs lead to larger particles and higher temperature, on the contrary, led to smaller particles. No significant influence on hydrodynamic diameter was observed for the other experimental parameters (Table S2). The significant positive effect of the monomer-to-IONP ratio on hydrodynamic diameter is consistent with expectations, as a higher ratio provides more polymerizable material per particle, facilitating the formation of thicker polymer coating.^35^ This leads to increased particle size due to the greater volume of polymer coating surrounding the iron oxide core. Interestingly, higher polymerization temperatures resulted in smaller hydrodynamic diameters. This may result from faster chain termination generating midchain radicals, which lead to complex reaction pathways and the formation of high-molecular-weight polymers, thereby affecting the termination mechanism and polymer coating structure.^36^ Conversely, lower temperatures likely promote slower, more continuous polymer growth and reduced termination,^37^ allowing thicker coatings to develop around each particle, thereby increasing the hydrodynamic size. The PDI seems to be consistent over the tested parameters ranging from 0.106 to 0.179.

**Figure 1:**
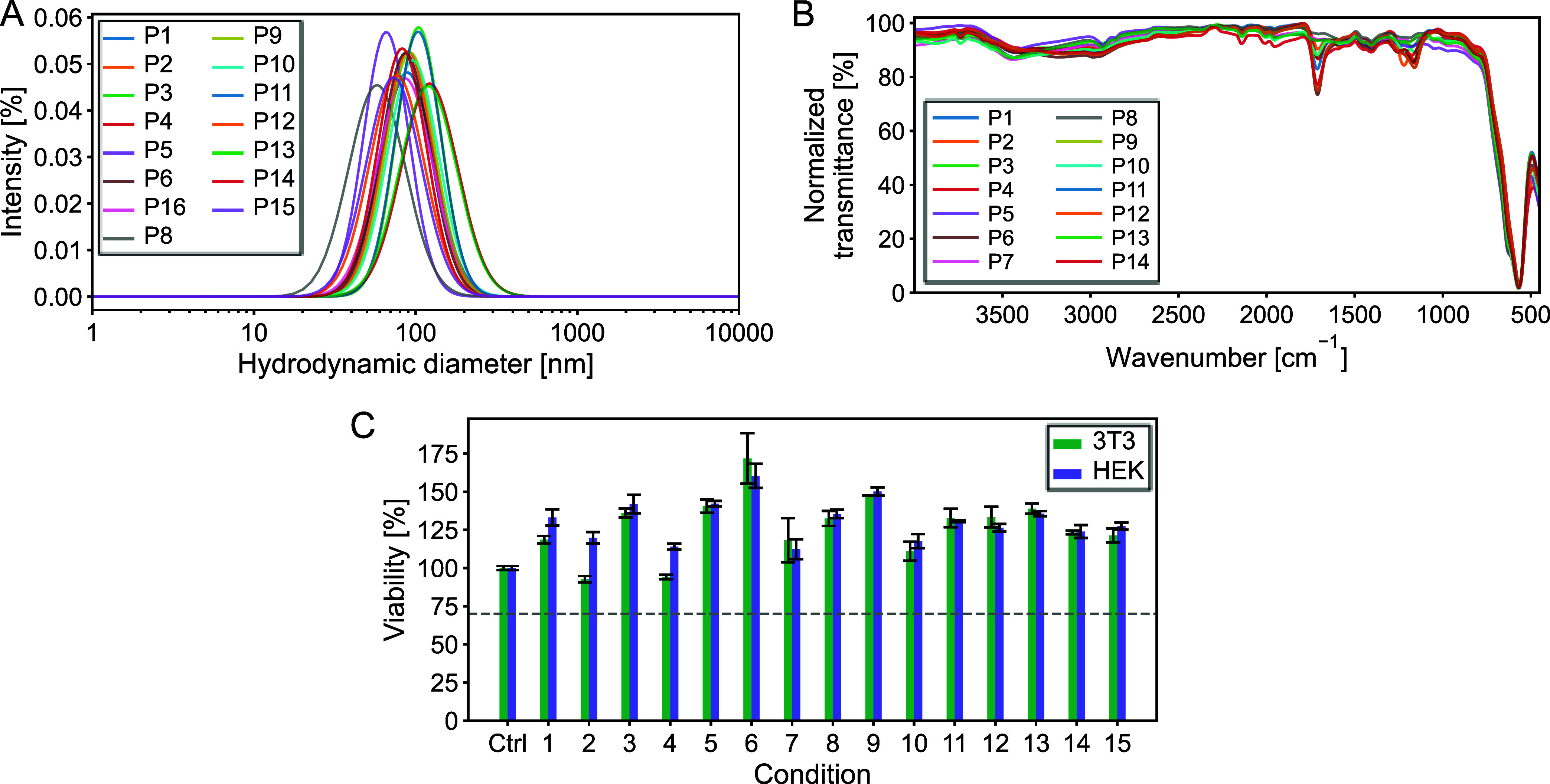
Influence of the synthesis parameters on properties of polymer-coated IONPs. Influence on (A) hydrodynamic diameter (DLS), (B) surface functional groups (FTIR), and (C) cytotoxicity. Hydrodynamic diameter was determined in ultrapure water (pH 7.2) and a particle concentration of 25 mg/L. Error bars represent the standard deviation from three independent measurements.

**Table 3:** Overview of the Influence of the Synthesis Parameters on Hydrodynamic Diameter (*z*-Average Diameter), PDI, ζ Potential, and Polymer Content (Weight Loss) in the Framework of a PBD^a^

condition	*Z*-average diameter [nm]	PDI	ζ potential [mV]	weight loss [%]
1	151	0.157	–32.2 ± 0.14	3.6 ± 0.14
2	142	0.130	–29.9 ± 0.0.64	2.7 ± 0.02
3	153	0.140	–35.3 ± 1.1	5.9 ± 0.06
4	146	0.109	–26.7 ± 0.50	3.3 ± 0.11
5	132	0.161	–30.9 ± 0.73	2.2 ± 0.22
6	222	0.179	–31.7 ± 0.27	3.8 ± 0.53
7	124	0.125	–27.8 ± 1.2	2.9 ± 0.16
8	128	0.164	–30.4 ± 0.81	3.6 ± 0.46
9	137	0.129	–32.2 ± 0.49	4.1 ± 0.27
10	164	0.106	–32.9 ± 2.0	4.3 ± 0.63
11	223	0.175	–33.1 ± 0.79	3.9 ± 0.09
12	105	0.110	–29.4 ± 0.34	4 ± 0.23
13	151	0.131	–34.5 ± 0.91	5.8 ± 0.44
14	171	0.166	–33.7 ± 0.76	2.6 ± 0.49
15	120	0.178	–31.6 ± 0.55	4.3 ± 0.85

a Hydrodynamic diameter and ζ potential were determined in ultrapure water (pH 7.2) and a particle concentration of 25 mg/L. Adsorption and desorption was performed in 25 mM PBS, pH 7.4. All values are presented as mean ± standard deviation from three independent measurements.

ATR-FTIR spectroscopy was used to assess the chemical composition of the ION@P­(AA-*co*-MAA), revealing key absorption bands associated with characteristic functional groups of acrylic acids and methacrylic acid (Figure 1B). These findings are consistent with previous reports.^23^ A prominent band at 565 cm^–1^ corresponds to (Fe–O) vibrations, indicative of the spinel structure of iron oxide,^38^ which could be also observed for bare IONPs (Figure S1B). Peaks at 1438 and 1593 cm^–1^, associated with symmetric and asymmetric stretching of deprotonated carboxylate groups [ν_s_(COO^–^) and ν_a_(COO^–^)], confirm the presence of carboxylic acids in their anionic form.^39^ Additionally, a peak at 1725 cm^–1^ is attributed to the (C=O) stretch of protonated carboxylic acid groups.^40^ Bands at 2856 and 2928 cm^–1^, corresponding to symmetric and asymmetric (C–H) stretching, are characteristic of methyl and methylene groups and suggest the presence of PAA, PMAA or both.^39,41^ Notably, under low monomer concentration conditions (P5, P8, P13), the FTIR signals appear less pronounced. In particular, for P5 and P8, this may be attributed to a thinner polymer coating, as supported by their smaller hydrodynamic diameters (Figure 1A) and lower weight loss upon heating (Table 1). Overall, the ATR-FTIR spectra suggest a successful coating in all conditions.

The ζ potential of the particles was measured at neutral pH (Table 3) and analyzed using OLS MLR. While the pH of bare IONPs was determined to be +10.3 mV (Table S1), the polymer-coated particles showed a negative ζ potential, regardless of the synthesis parameters (Table 3). The results revealed that both the ratio of monomers to IONPs and the polymerization temperature negatively influenced the ζ potential, with coefficients of −1.81 (*p* = 0.037) and −1.48 (*p* = 0.044), respectively (Table S3). These trends align with their effects on hydrodynamic diameter (Figure 1A and Table S2). Similarly, the negative impact of the monomer-to-IONP ratio on ζ potential may be explained by the formation of thicker polymer coatings at higher monomer contents,^35^ resulting in increased surface coverage by carboxylate groups. This can lead to a shift of the ζ potential toward more negative values. Likewise, higher polymerization temperatures may encourage the development of more compact polymer layers with more ionizable groups on the surface, also contributing to a more negative charge.

Regarding the weight loss, reflecting the amount of polymer associated with the nanoparticles, OLS MLR analysis showed that both the monomer-to-IONP ratio (β = 0.741, *p* = 0.007) and the polymerization temperature (β = 0.934, *p* = 0.004) had significant effects (Tables 3 and S4). A smaller, nonsignificant impact was observed for SDS concentration (*p* = 0.058), with only a minor coefficient of −0.375 (Table S4). As observed for hydrodynamic diameter and ζ potential (Figure 1A and Table 3), the significant positive effects of the monomer-to-IONP ratio and polymerization temperature on weight loss likely indicate increased polymer content on the nanoparticle surface. A higher monomer ratio provides more building blocks for shell formation,^35^ resulting in greater mass loss upon thermal decomposition. Likewise, elevated temperatures may enhance polymerization efficiency,^33^ yielding thicker polymer layers that contribute to increased weight loss during thermal analysis.

Although materials composed of PAA and PMAA have previously been approved by the EFSA,^27,28^ we wanted to investigate whether variations in the synthesis conditions of ION@P­(AA-*co*-MAA) influence the cytotoxicity of the resulting particles. As reported earlier,^23^ these particles did not negatively impact the viability of mouse embryonic fibroblasts (3T3) or HEK cells (Figure 1C) regardless of their synthesis conditions, suggesting good biocompatibility. However, it is important to note that this represents a relatively superficial assessment of cytotoxicity, focused only on short-term cellular viability and not accounting for long-term exposure, immune responses, or *in vivo* interactions. In practical applications, particularly those involving food contact, it is expected that the particles would be thoroughly removed after use. Nonetheless future studies should investigate additional safety parameters, including potential iron or polymer leaching under various pH and enzymatic conditions, as well as migration and stability testing according to food safety regulations.

In conclusion, the Plackett–Burman screening effectively identified the monomer-to-IONP ratio and polymerization temperature as the most influential parameters affecting the physicochemical characteristics of ION@P­(AA-*co*-MAA) particles, particularly hydrodynamic diameter, ζ potential, and weight loss. These findings were supported by ATR-FTIR spectroscopy, which confirmed successful polymer coating across all conditions and suggested variations in shell thickness linked to synthesis parameters. While other variables such as SDS concentration and initiator ratio showed minimal or no significant effects, the data highlight the critical role of polymerization conditions in tuning particle properties. Importantly, all tested formulations exhibited no apparent cytotoxicity in vitro, reinforcing the potential for safe application in food extraction contexts, even in scenarios involving trace particle residues.

### In-Depth Analysis Using CCD

As the PBD does not account for interaction effects or nonlinear responses, a subsequent face-centered CCD was employed to enable a more detailed optimization of the two most influential variables:^42^ the monomer-to-IONP ratio and polymerization temperature (Table 4). This design facilitated a systematic investigation of linear, quadratic, and interaction effects on key particle characteristics,^42^ with the aim of fine-tuning synthesis conditions for optimal performance in extracting histamine from foodstuff-mimicking buffers. As in the PBD, hydrodynamic diameter, ζ potential, and weight loss were analyzed. In addition, we assessed the binding capacity of particles synthesized under varying conditions.

**Table 4:** Experimental Conditions Used for the to Investigate the Effects of Synthesis Parameters on the Formation of Polymer-Coated IONPs in the Framework of the CCD^a^

condition	acrylic acid [μL]	methacrylic acid [μL]	APS [mg]	temperature [°C]
1	438	538	1753	70
2	51	63	206	60
3	438	538	1753	70
4	438	538	1753	70
5	824	1012	3300	70
6	824	1012	3300	60
7	824	1012	3300	80
8	51	63	206	70
9	51	63	206	80
10	438	538	1753	70
11	438	538	1753	60
12	438	538	1753	80
13	438	538	1753	70
14	438	538	1753	70

a Each condition represents a specific combination of factor levels, including central (0) and factorial (± 1) points.

OLS analysis of the DLS measurements (Figure 2A) revealed a significant dependence of the hydrodynamic diameter on the monomer-to-IONP ratio (β = 200.3, *p* = 0.001), with an even stronger effect observed for the quadratic term (β = 347.9, *p* = 0.001), indicating a nonlinear relationship (Tables 5 and S5). In contrast, polymerization temperature had no statistically significant effect on particle size under the tested conditions (Table S5). This suggests that increasing monomer content initially promotes polymer growth and attachment on the nanoparticle surface,^35^ thereby increasing particle size, but beyond a certain threshold, excessive monomer concentrations may lead to aggregation or insufficient interaction sites on the ION@P­(AA-*co*-MAA) surface,^43,44^ as reflected in the pronounced quadratic effect.

**Figure 2:**
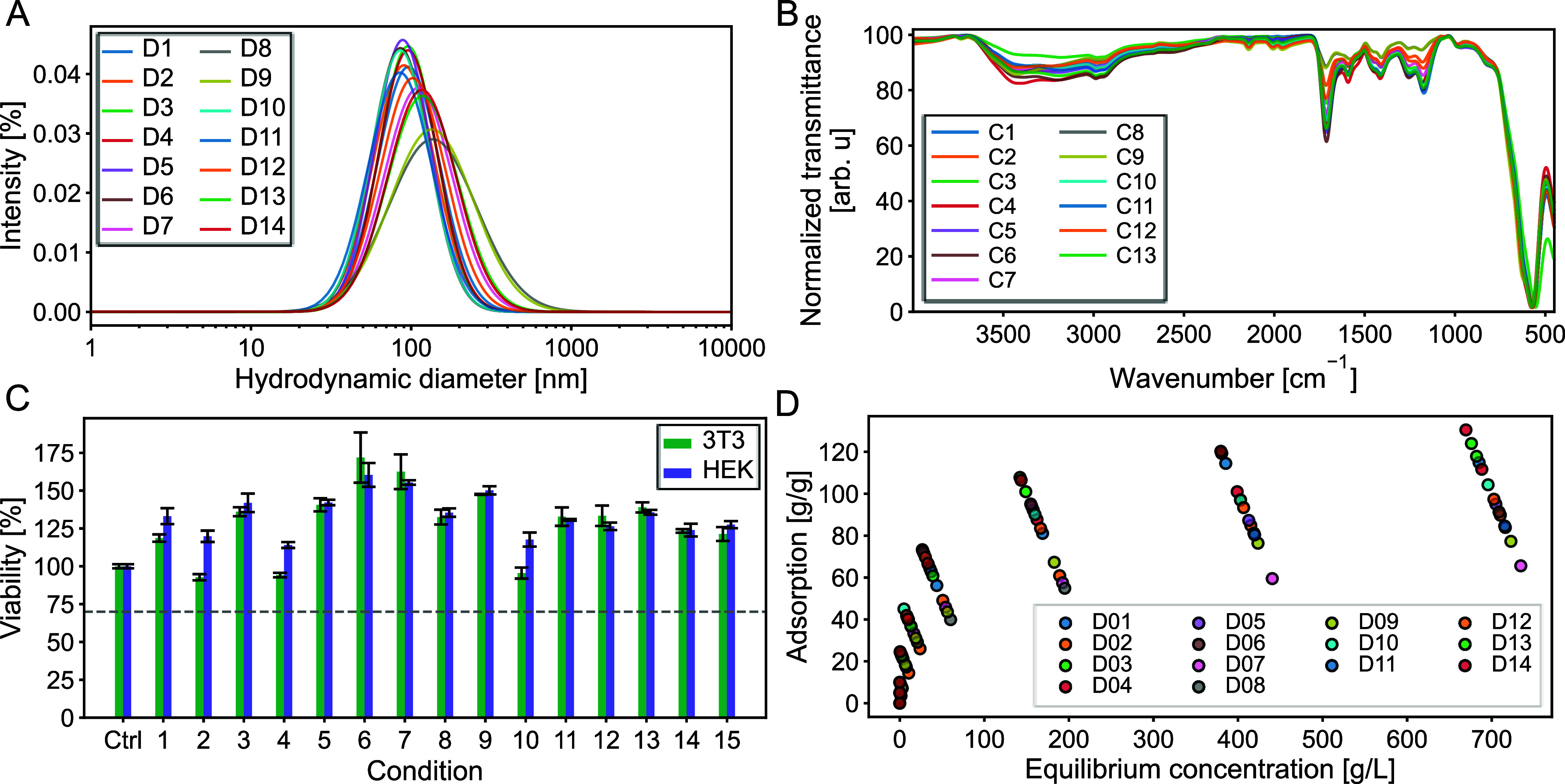
Influence of the synthesis parameters on properties of polymer-coated IONPs within the framework of a central composite design (CCD). Influence on (A) hydrodynamic diameter (DLS), (B) FTIR spectra, (C) cytotoxicity, and (D) histamine adsorption. Hydrodynamic diameter was determined in ultrapure water (pH 7.2) and a particle concentration of 25 mg/L. Adsorption and desorption was performed in 25 mM PBS, pH 7.4. Error bars represent the three independent measurements.

**Table 5:** Overview of Physicochemical Properties of Nanoparticles Synthesized According to the CCD.^a^

condition	*Z*-average diameter [nm]	PDI	ζ potential [mV]	weight loss [%]	adsorption [mg/g]	desorption [%]
1	239	0.220	–34.7 ± 1.3	4.6 ± 0.05	114.9 ± 2.16	4.4
2	307	0.271	–26.4 ± 0.56	3.4 ± 0.02	89.8 ± 3.08	20.7
3	220	0.232	–31.8 ± 0.58	4.5 ± 0.13	117.9 ± 1.30	6.2
4	261	0.245	–36.6 ± 0.86	4.4 ± 0.03	111.7 ± 1.40	8.6
5	196	0.191	–38.3 ± 1.65	5.0 ± 0.07	95.1 ± 1.46	8.6
6	801	0.496	–39.1 ± 0.80	4.2 ± 0.02	91.3 ± 0.168	8.7
7	703	0.433	–38.7 ± 0.34	5.9 ± 0.09	65.6 ± 8.75	26.5
8	380	0.293	–23.8 ± 1.0	3.3 ± 0.1	83.9 ± 0.472	16.9
9	352	0.275	–25.5 ± 0.27	4.5 ± 1.2	77.3 ± 1.89	22.1
10	210	0.217	–39.3 ± 1.1	4.5 ± 0.24	104.3 ± 2.01	9.9
11	205	0.185	–37.5 ± 0.16	3.9 ± 0.11	84.6 ± 1.44	12.9
12	199	0.191	–38.1 ± 0.71	5.1 ± 0.03	97.4 ± 1.19	15.3
13	182	0.175	–37.6 ± 1.9	4.5 ± 0.95	124 ± 1.01	9.7
14	188	0.187	–39.0 ± 0.87	4.5 ± 0.48	130.5 ± 1.27	10.4

a The table summarizes the hydrodynamic diameter (z-average diameter), PDI, ζ potential, polymer coating thickness (weight loss), adsorption and desorption for each synthesis condition in the framework of the CCD. Hydrodynamic diameter and ζ potential were determined in ultrapure water (pH 7.2) and a particle concentration of 25 mg/L. Adsorption and desorption was performed in 25 mM PBS, pH 7.4. All values are presented as mean ± standard deviation from three independent measurements.

The ζ potential measurements of the synthesized nanoparticles ranged from indicated generally high colloidal stability across the differently synthesis conditions (Table 5). OLS analysis revealed that among the tested factors, only the linear term of the monomer-to-IONPs ratio had a statistically significant effect on ζ potential (Table 6S), with a strong negative coefficient (−6.34, *p* = 0.001). This indicates that increasing the monomer concentration relative to the IONPs leads to a more negative surface charge, likely due to a higher presence of carboxy groups on the surface caused by the overall thicker polymer coating.^35^ In contrast, temperature, the squared terms, and the interaction term did not significantly affect the surface charge (Table S6).

ATR-FTIR spectra confirmed the presence of functional groups characteristic of acrylic acid and methacrylic acid, indicating successful polymer coating (Figure 2B), as already discussed in the previous section. Additionally, particles synthesized under varying conditions did not show any cytotoxic effects in the investigated cell lines (Figure 2C).

Weight loss was significantly influenced by the monomer-to-IONP ratio (Tables 5 and S7). The OLS model yielded a coefficient of 0.4579 (*p* = 0.001), indicating that higher monomer concentrations promote the formation of a thicker polymer coating on the nanoparticles. Similarly, increasing the temperature led to greater weight loss (β = 0.682, *p* = 0.001, Tables 5 and S7), suggesting enhanced polymer deposition on the IONPs. Additionally, the interaction between monomer concentration and temperature was significant (β = 0.127, *p* = 0.003, Tables 5 and S7), indicating a synergistic effect on polymer loading. As discussed for PBD, a higher monomer ratio supplies more building blocks for shell formation,^35^ while elevated temperatures likely accelerate polymerization,^33^ resulting in increased mass loss upon thermal decomposition

The histamine adsorption (Figure 2D) was significantly affected by the quadratic term of the monomer-to-IONP ratio (β = −25.95, *p* = 0.049) and temperature (β = −26.22, *p* = 0.010), while the linear monomer-to-IONP ratio also showed a notable, though not statistically significant, negative trend (β = −17.02, *p* = 0.070) (Figure 2D, Tables 5 and S8). The observed quadratic relationship between monomer-to-IONP ratio and histamine adsorption suggests that an optimal polymer composition exists for effective adsorption. At lower monomer concentrations, insufficient polymer coverage may lead to fewer available binding sites, reducing adsorption.^45^ Conversely, at very high monomer ratios, excessive polymer growth can cause denser coatings, potentially hindering histamine access to the carboxy groups or causing polymer collapse.^46^ The detrimental effect of elevated polymerization temperatures could result from faster polymerization rates that promote the formation of high-molecular-weight polymers, which in turn influence the termination process and alter the structure of the polymer coating.^36^ This can reduce the specificity or accessibility of binding sites by promoting more compact or irregular polymer networks, impairing histamine adsorption.

To further explore the structure–function relationships, the specific site density (SSD) and specific surface area (SSA) were approximated based on adsorption data and material characteristics. As expected, a positive association was observed between SSD and adsorption capacity (Figure S3A). This indicates that higher SSD contributes to increased histamine binding, thus, optimizing the density of accessible binding sites on the nanoparticle surface is critical for enhancing adsorption performance. Interestingly, a negative relationship was identified between SSA and SSD, suggesting that larger surface areas do not necessarily result in more functional binding sites (Figure S3B). Instead, high SSA may indicate a more porous or swollen polymer coating, which can reduce site accessibility.^47^ Similar studies have also already indicated that polymer structure and binding dynamics can disconnect particle size and therefore SSA from adsorption capacity.^48^ These findings imply that in the presented nanoparticle system, adsorption efficiency depends more on the accessibility of binding sites than on surface area alone.

Fitting of the adsorption data (Figure S2A-N) revealed a predominant prevalence of Sips isotherms, indicating primarily heterogeneous binding sites on the ION@P­(AA-*co*-MAA) nanoparticles, consistent with previous studies on ION@P­(AA-*co*-MAA) systems.^23^ This supports the presence of heterogeneous binding sites exhibiting varying stability and affinity.^49,50^ Such heterogeneity is expected given the nature of the P­(AA-*co*-MAA) polymer coating, where variations in polymer concentration, polymer–nanoparticle interactions, and coating density likely create surface regions with differing charge densities and binding characteristics.^51,52^ Interestingly, specific synthesis conditions influenced the adsorption behavior: conditions D4 and D12 (Figure S2D,L), synthesized with moderate monomer concentrations (Table 4), showed equally good fits with the Freundlich model, suggesting a more heterogeneous adsorption landscape, likely due to moderate polymer growth and variable coating density. In contrast, data from condition D5 (Figure S2E), which involved substantially higher monomer concentrations (Table 4), aligned better with the Langmuir model, indicative of more uniform, monolayer adsorption, possibly reflecting denser and more consistent polymer coating formation. Condition D6 (Figure S2F), synthesized with the same high monomer concentration as D5 but at a lower temperature (Table 4), showed comparable fits for both Langmuir and Sips models, indicating an intermediate adsorption behavior. The lower temperature may slow polymerization kinetics, resulting in less uniform coatings and a mixture of homogeneous and heterogeneous binding sites. Overall, these results demonstrate that subtle changes in synthesis parameters, particularly monomer concentration and temperature, modulate polymer coating morphology and heterogeneity, directly affecting the adsorption mechanism and distribution of binding sites on the nanoparticle surface.

In summary, the face-centered CCD enabled detailed optimization of monomer-to-IONP ratio and polymerization temperature, revealing complex nonlinear effects on nanoparticle size, surface charge, polymer content, and histamine adsorption capacity. While increased monomer content initially promoted polymer growth and binding site availability, excessive monomer concentrations and elevated temperatures adversely affected particle properties and adsorption efficiency, likely due to uncontrolled polymerization and structural changes in the polymer coating. Adsorption isotherm analysis further confirmed predominantly heterogeneous binding behavior, modulated by synthesis conditions, underscoring the critical role of polymer composition and processing parameters in tuning the performance of ION@P­(AA-*co*-MAA) nanoparticles for histamine extraction.

### Superparamagnetic Behavior and Separation Kinetics

After the identification of the most promising synthesis conditions for the adsorption of histamine, we wanted to characterize and investigate these optimized particles in more detail. First, the superparamagnetic properties of the particles were evaluated using vibrating sample magnetometry (VSM), as magnetic behavior plays a key role in separation efficiency. In particular, nanoparticles with higher saturation magnetization can be separated more easily using external magnetic fields.^53^ The resulting magnetization curve displayed the typical sigmoidal shape associated with superparamagnetic materials (Figure 3A), with no observable hysteresis or remanence at zero applied field.^54^ The particles reached a maximum magnetization of 56.8 emu/g, consistent with values reported in previous studies.^23,55^ While the overall magnetization behavior is characteristic of superparamagnetic materials, the slight slope observed at high magnetic fields indicates the presence of paramagnetic components. This may be attributed to residual paramagnetic iron ions, trapped water, or oxygen remaining in the sample.^56^

**Figure 3:**
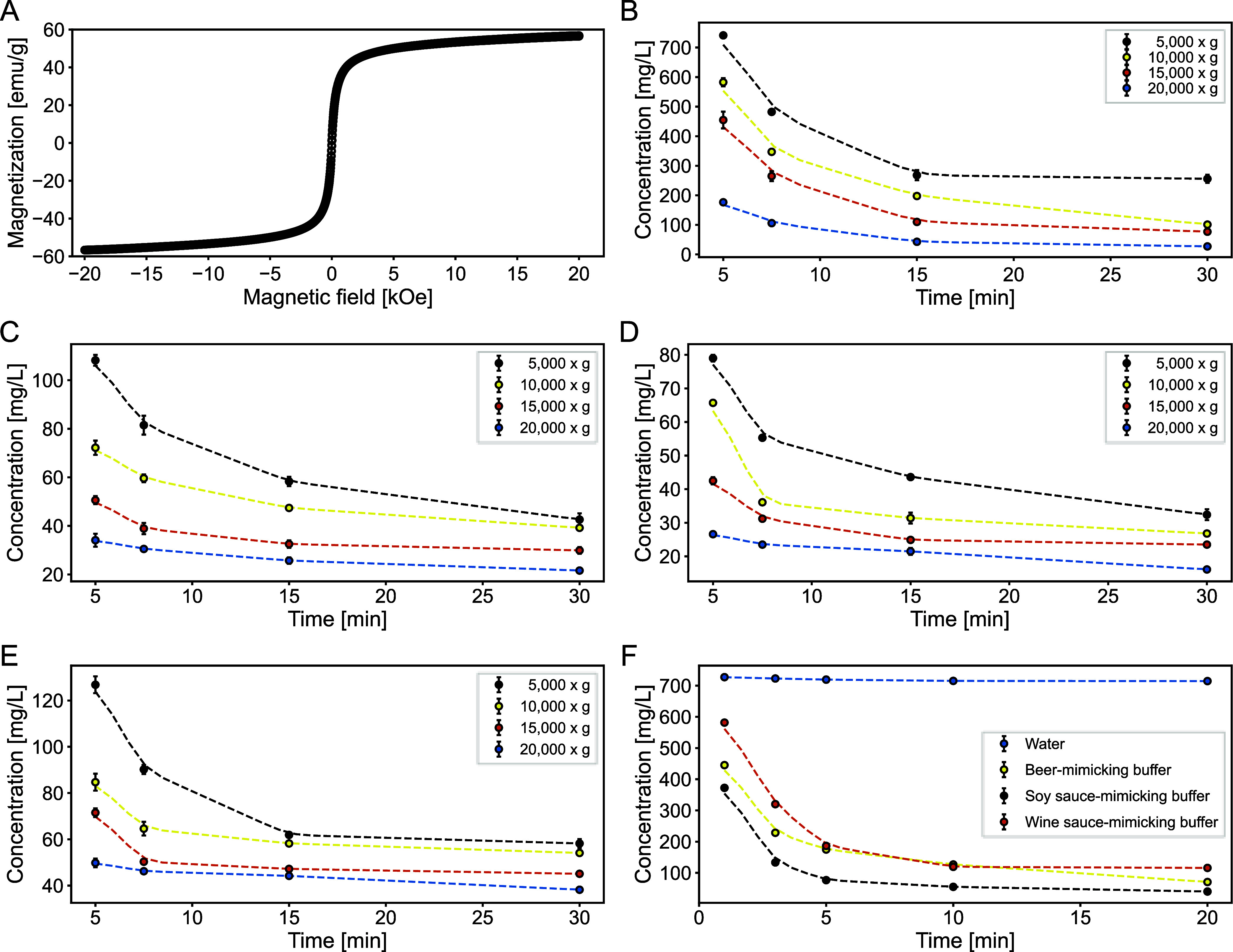
Magnetic properties and separation kinetics of optimized ION@P­(AA-*co*-MAA). (A) Superparamagnetic behavior determined by VSM. Separation kinetics by centrifugation with the indicated speed in (B) water, (C) beer- (pH 4.4), (D) soy sauce- (pH 5.0), and (E) wine-mimicking buffer (3.8). (F) Separation kinetics by magnetism in water, soy sauce-, beer-, and wine-mimicking buffer. All values are presented as mean ± standard deviation from three independent measurements.

Next, the separation kinetics by centrifugation were evaluated in water and various food-mimicking buffers by measuring the residual concentration of ION@P­(AA-*co*-MAA) in the supernatant after centrifugation at different speeds. The chosen buffers, beer, wine, and soy sauce mimics, represent simplified matrices designed to approximate the physicochemical conditions of histamine-rich foods. These are relevant targets for biogenic amine extraction due to their known high histamine content.^57–59^ Separation rate kinetics were determined for each centrifugation speed tested. Since particle separation in water was inefficient (Figure 3B), detailed kinetic analysis was not conducted for these samples. Overall, separation in water was the least effective among all tested media, both for centrifugation and magnetic separation methods (Figure 3B,F), compared to the food-mimicking buffers.

In beer-mimicking buffer, optimal separation was achieved by centrifugation at 20,000*g* for 30 min (Figure 3C). After this period, only 21.6 mg/L of particles remained in suspension, corresponding to just 2.2% of the initial particle concentration, indicating highly efficient sedimentation. At all tested centrifugation speeds, the separation kinetics were best described by a double exponential function (Figure S4A–D), suggesting a two-phase sedimentation process.^60^ The initial fast phase likely corresponds to the rapid sedimentation of larger particles and/or preformed aggregates due to their higher mass and lower resistance to centrifugal force. This is followed by a slower phase, in which smaller or more dispersed particles settle gradually, possibly due to increased drag forces or stabilizing interactions with the buffer components.^61^ The presence of two distinct kinetic phases may also reflect heterogeneity in particle size distribution.^62^ The improved separation efficiency in beer-mimicking buffer compared to water may be attributed to the presence of salts in the buffer. These components can screen electrostatic repulsion between particles, promoting aggregation and thereby enhancing sedimentation, particularly in the early phase of the separation process.^63^

For the soy sauce-mimicking buffer, similar to the beer-mimicking system, the most effective particle separation was achieved by centrifugation at 20,000*g* for 30 min (Figure 3D). Postcentrifugation, only 16.1 mg/L of ION@P­(AA-*co*-MAA) remained detectable by spectrophotometry, corresponding to just 1.6% of the initial particle concentration in suspension. This improved separation relative to the beer-mimicking buffer is likely due to the higher salt concentration in soy sauce, which can effectively shield the surface charge of the nanoparticles, thereby reducing electrostatic repulsion and promoting aggregation that enhances sedimentation.^63^ As observed for the beer-mimicking buffer, separation in the soy sauce-mimicking buffer also follows a two-phase sedimentation process, with an initial rapid sedimentation of larger particles or aggregates (Figure S5A–D), followed by a slower phase where smaller particles gradually separate.

In the wine-mimicking buffer, centrifugation at 20,000*g* for 30 min resulted in 38.2 mg/L of ION@P­(AA-*co*-MAA) remaining in the supernatant (Figure 3E), corresponding to a removal efficiency of approximately 96.2% from the initial ION@P­(AA-*co*-MAA). The separation kinetics were well described by a double exponential model across all tested centrifugation speeds (Figure S6A–D). These results demonstrate that, similar to the other food-mimicking buffers, particle separation in the wine-mimicking buffer follows a two-phase sedimentation process influenced by centrifugation speed.

Magnetic separation of ION@P­(AA-*co*-MAA) was investigated in water and the same buffers as before (Figure 3F). In water, the particles exhibited only a weak tendency to sediment over 20 min, indicating poor separation efficiency in the absence of salts that could facilitate aggregation or magnetophoretic mobility.^64^ After 20 min, only 28.5% of the initial particle concentration were removed from the supernatant. In contrast, magnetic separation was markedly enhanced in beer-, soy sauce-, and wine-mimicking buffers, all of which resulted in rapid decreases in supernatant concentrations over time. Among the tested kinetic models, the double exponential model consistently provided the best fit across all buffer conditions (R2 ≥ 0.999). For instance, in soy sauce buffer, an initial rapid removal phase (*k*_1_ = 0.76 min^–1^) removed the majority of particles within the first 5 min, followed by a slower phase (*k*_2_ = 0.03 min^–1^), likely representing the sedimentation of smaller or less magnetically responsive particles. First-order and Elovich models yielded negative *R*2 values, suggesting they are unsuitable for describing this process. The pseudo-second-order model showed moderate agreement only in soy sauce (*R*2 = 0.88) and wine (*R*2 = 0.79), indicating some relevance of surface-based interactions but overall inferior fit compared to the double exponential model. The observed enhancement in separation in mimicking buffers can be attributed to the presence of salts, which promote particle aggregation and increase the effective magnetic susceptibility of clusters, thereby accelerating magnetic separation. These insights highlight the importance of environmental matrix composition for efficient nanoparticle recovery, with potential implications for applications in food-related systems.

### Application of ION@P­(AA-*co*-MAA) in Food Matrix-Mimicking Buffers

Given the promising separation performance of ION@P­(AA-*co*-MAA) under both centrifugation and magnetic conditions, particularly in beer- and soy sauce-mimicking buffers, we next investigated their adsorption capacity for histamine in beer-, soy sauce-, and wine-mimicking environments.

In beer-mimicking buffer, a maximum adsorption capacity of 29 ± 1.4 mg/g was achieved at the highest tested histamine concentration (Figure 4A and Table 6). Consistent with previous findings for these particles,^23,63,64^ the adsorption behavior follows a Sips isotherm model (Figure 4A), suggesting a heterogeneous surface with binding sites that vary in affinity and stability.^49,50^ As outlined in the previous section, such heterogeneity is anticipated due to the nature of the P­(AA-*co*-MAA) polymer coating, where differences in polymer concentration, polymer–nanoparticle interactions, and coating density likely result in surface regions with varying charge densities and binding properties.^51,52^

**Figure 4:**
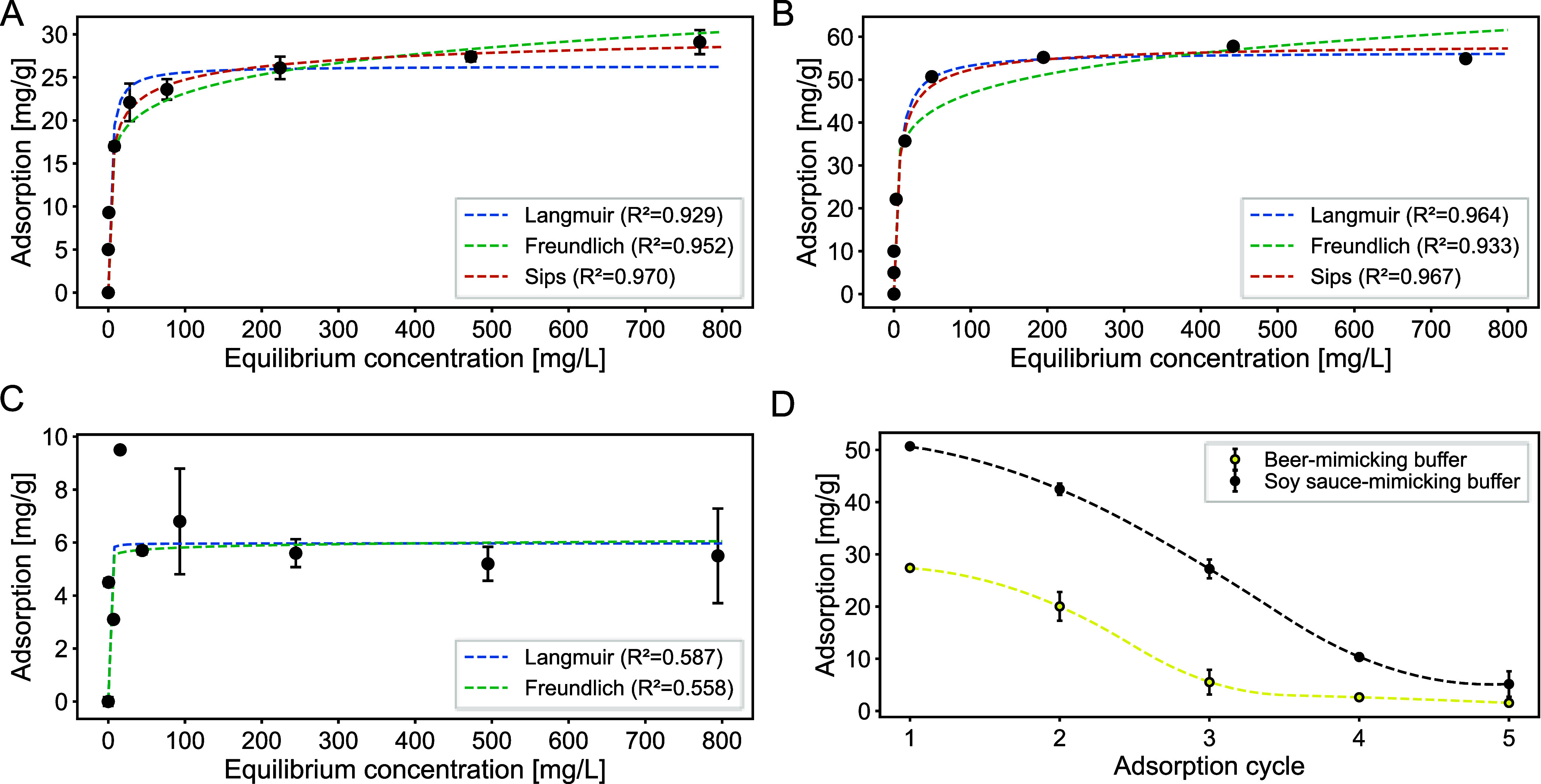
Histamine adsorption performance of ION@P­(AA-*co*-MAA) in food-mimicking buffers. Adsorption isotherms with corresponding model fits for histamine in (A) beer- (pH 4.4), (B) soy sauce- (5.0), and (C) wine-mimicking buffers (3.8). (D) Histamine adsorption after the indicated number of adsorption–desorption cycles in beer- and soy sauce-mimicking buffers. All values are presented as mean ± standard deviation from three independent measurements.

**Table 6:** Maximum Histamine Adsorption by ION@P­(AA-*co*-MAA) at an Initial Concentration of 800 mg/L in Beer- (pH 4.4), Soy Sauce- (pH 5.0), and Wine-Mimicking Buffer (pH 3.8)^a^

buffer	adsorption [mg/g]
beer-mimicking buffer	29 ± 1.4
soy sauce-mimicking buffer	55 ± 0.39
wine-mimicking buffer	5.5 ± 1.8

a Values represent mean ± standard deviation from three independent measurements.

In soy sauce-mimicking buffer, the maximum adsorption capacity reached 55 ± 0.39 mg/g (Figure 4B and Table 6), drastically higher than in beer-mimicking buffer. As with previous measurements, the adsorption data followed a Sips isotherm, which further confirms that the polymer-coated nanoparticles have a heterogeneous surface. The higher adsorption observed here may also be influenced by the higher ionic strength and presence of salts in the soy sauce-mimicking buffer, which could potentially induce a more favorable conformation of the polymer network.^63^

In contrast, adsorption in the wine-mimicking buffer was substantially lower, with a maximum of only 5.5 ± 1.8 mg/g (Figure 4C and Table 6). Fitting the data to a Sips isotherm was not possible, and the low *R*2 values indicate poor model agreement. This result is most likely due to the acidic pH of the buffer (3.8), which is below the p*K*_a_ of the polymer coating.^65^ At this pH, the majority of carboxyl groups are protonated, eliminating their negative charge and thus weakening or entirely suppressing the electrostatic attraction between the polymer and the positively charged histamine molecules. Additionally, the low pH could destabilize the polymer structure, further hindering adsorption.^63^

In summary, the adsorption performance of the polymer-coated nanoparticles is strongly influenced by the buffer composition, with ionic strength and pH playing key roles. While high adsorption in soy sauce- and beer-mimicking conditions confirms the presence of heterogeneous, high-affinity binding sites, acidic environments such as wine severely limit binding capacity. These findings underscore the importance of electrostatic interactions and polymer charge state in dictating adsorption efficiency.

To evaluate the reusability of ION@P­(AA-*co*-MAA), five consecutive adsorption–desorption cycles were performed in beer- and soy sauce-mimicking buffers. As summarized in Table 7, a progressive decline in adsorption capacity was observed in both buffers, though the kinetics and magnitude of the loss varied markedly between conditions (Figure 4D).

**Table 7:** Adsorption and Recovery of Histamine after Repeated Use of ION@P­(AA-*co*-MAA)^a^

adsorption/desorption cycle	1	2	3	4	5
beer-mimicking buffer	27 ± 0.40 (81%)	20 ± 2.7 (48%)	5.5 ± 2.4 (69%)	2.6 ± 0.59 (3%)	1.5 ± 0.21
soy sauce-mimicking buffer	51 ± 0.11 (93%)	42 ± 1.1 (85%)	27 ± 1.8 (79%)	10 ± 0.56 (29%)	5.2 ± 2.4

a Adsorption capacity [mg/g] after *x* cycles, with the corresponding recovery shown in brackets [%], in beer- (pH 4.4) and soy sauce-mimicking buffers (pH 5.0). Values in represent mean ± standard deviation from three independent measurements.

In the beer-mimicking buffer, the adsorption capacity dropped rapidly across cycles (Figure 4D and Table 7). After an initial capacity of 27 ± 0.40 mg/g, the second cycle showed a notable decrease to 20 ± 2.7 mg/g. The adsorption dropped further to 5.5 ± 2.4 mg/g in the third cycle, with minimal adsorption observed in the fourth (2.6 ± 0.59 mg/g), and fifth cycles (1.5 ± 0.21 mg/g). This steep decline most likely reflects a combination of irreversible histamine adsorption to high-affinity sites, polymer matrix rearrangement, and fouling after repeated use.^66^ This fact is especially evident when looking at the percentage of recovery after each adsorption cycle (Table 7), indicating that only a small fraction of histamine could be successfully desorbed. In relatively low-ionic-strength environments like the beer buffer, the polymer chains may adopt a more expanded or flexible conformation that facilitates initial adsorption but becomes less stable after desorption, reducing the availability and accessibility of functional sites in subsequent cycles.^63,66^

In contrast, the soy sauce-mimicking buffer supported a more gradual decline in adsorption capacity (Figure 4D and Table 7). Starting from 51 ± 0.11 mg/g, adsorption remained relatively high during the second (42 ± 1.1 mg/g) and third cycles (27 ± 1.8 mg/g), before declining to 10 ± 0.56 mg/g and 5.2 ± 2.4 mg/g in the final two cycles. The sustained performance in this buffer may be attributed to the high ionic strength, which may stabilize the polymer coating and maintain a more favorable binding site geometry over repeated use.^63^ This fact is also evident in the higher percentage of recovery compared to the beer-mimicking buffer (Table 7). Additionally, partial reversibility of interactions and resistance to structural collapse may contribute to improved binding site longevity.

These results highlight both the promise and the limitations of the presented system. While the polymer-coated nanoparticles demonstrate reusability under certain conditions, particularly in salt-rich matrices, binding site saturation, incomplete desorption, or material fatigue significantly limit performance in other settings. Improving desorption protocols or incorporating regenerable binding motifs may offer solutions to extend the operational lifespan of these materials for repeated use. These results highlight the promising potential of ION@P­(AA-*co*-MAA) nanoparticles for histamine removal, it is important to note that the current evaluations were conducted primarily in simplified, optimized buffer systems. Further investigation is required to assess their performance and stability in complex, real-world food matrices where factors such as protein content, competing analytes, and matrix heterogeneity may affect adsorption efficiency and particle recovery.

## Conclusions

This study presents a comprehensive evaluation and optimization of ION@P­(AA-*co*-MAA) as efficient, reusable adsorbents for histamine extraction in food-mimicking environments. Through an initial Plackett–Burman screening, the monomer-to-IONP ratio and polymerization temperature were identified as critical synthesis parameters significantly influencing nanoparticle size, surface charge, and polymer coating thickness. Subsequent detailed optimization using a face-centered CCD revealed complex, nonlinear dependencies of these factors on physicochemical properties and histamine binding capacity, underscoring the importance of precise control over polymer composition and synthesis conditions.

The optimized nanoparticles exhibited typical superparamagnetic behavior, enabling rapid and efficient separation by both centrifugation and magnetic methods, particularly in high ionic strength buffers. Adsorption experiments demonstrated histamine binding capacity in food-mimicking environments, with maximum adsorption reaching 55 mg/g in soy sauce-mimicking buffer, attributed to favorable polymer conformations and electrostatic interactions under elevated ionic strength. Conversely, the acidic wine-mimicking buffer drastically reduced histamine adsorption, underscoring the pH limitation of the particle system.

Notably, the adsorption capacity achieved here compares well with those of other histamine-binding materials, all of which were tested under optimized conditions. For example, Fe_3_O_4_@Agarose@Silica nanoparticles have achieved up to 178 mg/g in human serum.^67^ Natural adsorbents such as zeolites typically exhibit much lower capacities, such as 10.2 mg/g for zeolithe.^68^ Biological methods involving lactic acid bacteria have demonstrated up to 57% histamine removal under controlled conditions.^69^ However, these approaches may face challenges with scalability, batch-to-batch variability, and regulatory acceptance for industrial use. In contrast, the polymer-coated IONPs developed in this study provide a practical balance between performance, biocompatibility, and magnetic recoverability, supporting their potential for scalable deployment in histamine-sensitive food applications.

To assess operational practicality, reusability assessments revealed a significant decline in adsorption capacity over multiple cycles, particularly in low ionic strength media such as the beer-mimicking buffer, where capacity dropped from 27 mg/g initially to less than 2 mg/g by the fifth cycle. This rapid decline likely results from irreversible histamine binding, polymer structural changes, and fouling. These findings underscore a critical limitation in operational durability that must be addressed in future studies. Optimizing desorption protocols or developing effective regeneration strategies could enhance particle reusability and sustain adsorption efficiency, especially under low ionic strength conditions.

Cytotoxicity testing indicated low acute toxicity; however, this assessment was limited to short-term cellular viability and did not account for long-term exposure, immune responses, or *in vivo* effects. Given the intended food-related applications where particles would be removed after use, thorough future studies are essential to guarantee long-term safety which are in line with food safety regulations to ensure consumer safety.

While these results highlight the promising potential of ION@P­(AA-*co*-MAA) nanoparticles for histamine removal, it is important to note that the current evaluations were conducted primarily in simplified, optimized buffer systems. Further investigation is required to assess their performance and stability in complex, real-world food matrices where factors such as protein content, competing analytes, and matrix heterogeneity may affect adsorption efficiency and particle recovery.

Overall, this work establishes ION@P­(AA-*co*-MAA) nanoparticles as promising candidates for efficient histamine removal in the production of low-histamine foodstuffs and food safety applications, combining tunable surface chemistry, superparamagnetic responsiveness, and practical reusability. Future efforts focusing on optimized desorption protocols, regeneration strategies, and real matrix testing will be critical to fully realize their potential for practical food monitoring and purification processes.

## Experimental Section

### Materials

Acrylic acid (≥99%, stabilized with hydroquinone monomethyl ether), hydrochloric acid (37%), iron­(II)­chloride tetrahydrate (98%), iron­(III)­chloride anhydrous (97%), methacrylic acid (≥99%, stabilized with hydroquinone monomethyl ether), phosphotungstic acid hydrate, sodium dodecyl sulfate (≥99%,), and sodium hydroxide pellets (≥97%) were purchased from Sigma-Aldrich Handels GmbH (Vienna, Austria). Ammonium persulfate (≥98%) was purchased from Carl Roth GmbH + Co. KG (Karlsruhe, Germany). Histamine was purchased from TCI Deutschland GmbH (Eschborn, Germany). Dulbecco’s Modified Eagle Medium (DMEM, 4.5 g/L glucose, 2 mM l-glutamine), fetal bovine serum (FBS), penicillin-streptomycin (10.000 U/mL), and XTT assay kit were purchased from Thermo Fisher Scientific GmbH (Vienna, Austria). Normocin and HEK-Blue Selection were purchased from InvivoGen SAS (Toulouse, France).

### Synthesis of IONPs

Superparamagnetic IONPs were synthesized via coprecipitation, following a previously established and published protocol.^70^ For the synthesis, 14.45 g (361.5 mmol) sodium hydroxide was dissolved in 200 mL degassed ultrapure water. Separately, 10.4 g (64 mmol) anhydrous FeCl_3_ and 7.0 g (35.2 mmol) FeCl_2_·4·H_2_O were dissolved in 80 mL degassed ultrapure water. The iron salt solution was then slowly added to the sodium hydroxide solution under constant mechanical stirring (150 rpm), and the reaction was allowed to proceed for 30 min at RT. The resulting IONPs were transferred to a glass flask and washed 15 times with degassed ultrapure water using magnetic decantation, until the pH reached ≥ 7.6. The nanoparticles were then resuspended in 200 mL of degassed ultrapure water and stored at 4 °C. The mass concentration was determined gravimetrically by drying the particles overnight at 60 °C.

### Polymer Coating of IONPs

The polymer coating of IONPs was carried out in accordance with a previously published protocol.^23^ For both the PBD and CCD formulations, 100 mg IONPs were placed in a glass flask with a ported cap and filled to a final volume of 100 mL using degassed ultrapure water. The suspension was redispersed using an ultrasonic processor (Model 120 Sonic Dismembrator, Fisherbrand) for 5 min (applying a cycle of 10 s on and 15 s off at 30% amplitude). The suspension was transferred to an ultrasonic bath and degassed under vacuum for 30 min. The flask was then evacuated with nitrogen and the suspension subjected to nitrogen bubbling for an additional 20 min. Specific synthesis parameters for each condition are detailed in Table 2 (PBD) and Table 4 (CCD). Under continuous stirring at 250 rpm, either the specified amount of sodium dodecyl sulfate (for PBD) or 2.5 mg (for CCD) was added, and the suspension was heated to the target temperature in a water bath. Once the desired temperature was reached, the appropriate number of monomers was introduced. After monomer addition, the mixture was allowed to equilibrate for 45 min. Subsequently, ammonium persulfate (APS) was added to initiate polymerization, maintaining a constant molar ratio of 0.6025 across all conditions; the APS was dissolved in 4 mL of ultrapure water prior to addition. The reaction was maintained at a constant temperature with continuous stirring for the specified duration (for PBD) or for 30 min if not otherwise indicated. Throughout the process, the headspace of the reaction vessel was continuously purged with nitrogen. Upon completion of the polymerization, the polymer-coated nanoparticles were separated using magnetic decantation and washed thoroughly with a total of 2 L ultrapure water. The nanoparticles were then redispersed in ultrapure water and stored at 4 °C. The mass concentration was determined gravimetrically by drying the particles overnight at 60 °C.

### Physicochemical Characterization of Polymer-Coated IONPs

The hydrodynamic diameter of the nanoparticles was measured using dynamic light scattering (VASCO Flex, Cordouan Technologies SAS). For this, each nanoparticle formulation was diluted in ultrapure water (pH ∼ 7.2) to a final concentration of 25 mg/L. The diluted samples were transferred into disposable plastic cuvettes (Brand GmbH + CO KG) and analyzed at room temperature. ζ-potential measurements were carried out using a Zetasizer Nano ZS (Malvern Panalytical, Ltd.) at 25 °C, with particle suspensions also adjusted to a concentration of 25 mg/L in ultrapure water at pH 7.2.

#### Attenuated Total Reflectance Fourier-transform Infrared Spectroscopy (ATR-FTIR)

For analysis of the chemical composition using ATR-FTIR, 1 μL of nanoparticle suspension was placed on the ATR crystal and the liquid was evaporated by the application of cold air. The data were recorded (4 scans) using a UATR-FTIR (Spectrum Two, PerkinElmer, Inc.) equipped with a diamond ATR crystal and DTGS detector at room temperature.

#### TEM

The morphology and size of the IONPs were analyzed using a TEM (Tecnai G20, FEI Company) operated at 120 kV. Prior to imaging, the nanoparticles were diluted to 10 mg/L in ultrapure water and dispersed via ultrasonication at 75% amplitude. To polymer-coated IONPs, phosphotungstic acid was added to reach a final concentration of 2% and incubated for 1 min. Afterward, a drop of the suspension was placed onto glow-discharged, carbon-coated copper grids (200 mesh, PELCO). Imaging was performed using a CCD camera (BM-Ultrascan 1000P, Gatan, Inc.).

#### Vibrating Sample Magnetometry

Magnetic measurements were conducted using a vibrating sample magnetometer (Lake Shore Cryotronics, Inc.) equipped with an EM7-CSB magnet capable of generating magnetic fields up to 3.2 T. Prior to measurement, the samples were freeze-dried. Data was continuously collected at 293 K across nine magnetic field intervals, beginning from 0 Oe.

### Adsorption Isotherms

For CCD analysis, adsorption isotherms were carried out in 0.25 M PBS (pH 7.4). Histamine was initially dissolved in 0.5 M PBS (pH 7.4) at a concentration of 4 g/L and subsequently diluted to 0.5 g/L and 50 mg/L through serial dilution. ION@P­(AA-*co*-MAA) particles were prepared at a working concentration of 2 g/L. In 1.5 mL Eppendorf tubes, 500 μL particle suspension was combined with appropriate volumes of histamine stock solutions (4 g/L, 0.5 g/L, or 50 mg/L) to achieve a final particle concentration of 1 g/L. Final histamine concentrations in the samples were set at 2, 1, 0.5, 0.25, 0.1 g/L, and 50, 25, 10, 5 mg/L. The volume of each sample was adjusted to 1 mL using 25 mM PBS. The mixtures were incubated for 3 h at 24 °C on an orbital shaker set to 1000 rpm. After incubation, magnetic separation was performed for 10 min, and 600 μL of the supernatant was collected for analysis. To wash the particles, the remaining supernatant was discarded, and the nanoparticle pellet was resuspended in 1 mL of 25 mM PBS. The suspension was shaken at 24 °C and 1000 rpm for 10 min, followed by magnetic separation. Another 600 μL of supernatant was collected (wash 1). This washing step was repeated once more to obtain wash 2. All collected samples (adsorption, wash 1, and wash 2) were analyzed using a UV spectrophotometer (Shimadzu, UV-1800) with a quartz cuvette (Hellma, 100-QS, 10 mm) at 210 nm. The amount of histamine adsorbed onto the nanoparticles was determined by subtracting the histamine content in the supernatant from the initial amount added. Desorption during the washing steps was also quantified, and cumulative desorption was reported as a percentage. For the adsorption isotherm in beer-, soy sauce-, and wine-mimicking buffers, the following solutions were used: 10 mM NaCl, 5 mM KCl, 5% ethanol, and pH adjusted to 4.4 (beer-mimicking buffer); 2.5 M NaCl, pH adjusted to 5.0 (soy sauce-mimicking buffer); 30 mM NaCl, 12% ethanol, and pH adjusted to 3.8. For the evaluation of the reusability of the nanoparticles, the histamine-loaded particles were redispersed in 1 mM HCl (pH ≈ 3) for 2 h on an orbital shaker at 24 °C and 1000 rpm to remove the histamine from the particles. Afterward, the nanoparticles were washed with the respective buffer and incubated for 3 h in 100 mg/L histamine in the respective buffer.

### Separation Behavior

The separation behavior of IONPs was assessed in aqueous media and in food matrix-mimicking buffer solutions. Experiments were conducted at a sample volume of 1 mL with an ION@P­(AA-*co*-MAA) concentration of 1 g/L. For centrifugation-based separation, samples were subjected to varying speeds and durations: 5000, 10,000, 15,000, and 21,000 rpm for 5, 15, or 30 min each. For magnetic separation, a constant magnetic field was applied for durations of 1, 3, 5, 10, and 20 min. After separation, the supernatant was collected, and particle concentration was quantified using a UV spectrophotometer (Shimadzu, UV-1800) at 360 nm in a quartz cuvette (Hellma, 100-QS, 10 mm). To analyze the separation kinetics, the percentage of particles remaining in suspension was measured over time at fixed RCF or magnetic conditions. These data were fit using a single-exponential decay model:

$$C_{t}=C_{0}\times e^{-\mathrm{kt}}$$

where *C*_*t*_ is the concentration at time *t*, *C*_0_ is the initial concentration, and *k* is the separation rate constant. Values of *k* were extracted for each condition to compare separation efficiency across different forces and time scales.

### Cytotoxicity Assay in Mammalian Cells

Cytotoxicity of IONPs was verified with an XTT cell proliferation assay (CyQUANT XTT Cell Viability Assay, Invitrogen) in HEK-Blue TLR4 and 3T3-L1 mouse fibroblasts. The assay was conducted following the instructions outlined in the manual. HEK cells were seeded at a density of 6,000 cells in 100 μL of medium per well, while 3T3 cells were seeded at a density of 1000 cells in 100 μL of medium per well. For the cultivation of 3T3 and HEK-Blue TLR4 cells, DMEM was supplemented with 10% (v/v) heat inactivated FBS and1% penicillin-streptomycin (100 μg/mL). For HEK-Blue TLR4 cells, the medium was additionally supplemented with 1 mL Normocin (100 μg/mL) and 2 mL HEK-Blue Selection per 500 mL of medium. Both cell types were plated onto a 96-well plate. The cells were incubated in the respective growth medium at 37 °C and 5% CO2 for 48 h to reach a confluence close to 90%. 50 μL of the reconstituted XTT mixture was added to the cells and mixed well before incubation for 4 h at 37 °C protected from light. The absorbance was measured at 450 and 660 nm with a UV–vis spectrophotometer (PowerWave Select X, Bio-Tek Instruments, Inc.).

### Calculation of SSA and SSD

he specific surface area (SSA) of the polymer-coated IONPs was estimated based on the total particle diameter (*d*_total_) obtained from DLS and an assumed core size of 10 nm (from TEM). The volumes of the core (*V*_core_) and the total particle (*V*_total_) were calculated assuming spherical geometry. The polymer coating volume (V_shell_) was obtained by subtraction: *V*_shell_ = *V*_total_ – *V*_core_. The composite density (ρ_composite_) was calculated using a volume-weighted average of the core and shell densities, where ρ_cor_ e = 5.2 g/cm^3^ (Fe_3_O_4_) and ρ_shell_ = 1.2 g/cm^3^ (polymer):

$$\rho_{\mathrm{composite}}=\frac{\rho_{\mathrm{core}}\times V_{\mathrm{core}}+\rho_{\mathrm{shell}}\times V_{\mathrm{shell}}}{V_{\mathrm{to}t\mathrm{al}}}$$

The SSA was calculated using

$$SSA=\frac{6}{\rho_{composite}\times d_{total}}$$

The SSD was calculated by dividing the maximum number of adsorbed histamine molecules per gram of nanoparticle (from adsorption isotherms) by the estimated SSA:

$$SSD=\frac{adsorbed\;molecules}{SSA}$$

### Data Analysis and Visualization

The data was analyzed and visualized in Python 3.12.0. the analysis employed the SciPy^71^ and statsmodels^72^ libraries. Outliers were identified using Cook’s distance, with the threshold set at 4/number of observations. For the PBD, the final model was determined by ordinary least-squares multiple linear regression. Diagnostic evaluation of model assumptions included an assessment of multicollinearity using the variance inflation factor (VIF). For the CCD, a quadratic multiple linear regression model with interaction terms was applied as part of response surface modeling. Model diagnostics were also performed using VIF to assess multicollinearity. The final regression models were then used to identify and summarize the significant factors influencing the outcome. Data visualization was performed with the Matplotlib^73^ package.

## Supplementary Material



## Notes

Statistical analysis of synthesis factors, histamine adsorption isotherms, and nanoparticle separation kinetics in various buffers (PDF)

## Abbreviations

APS: ammonium persulfate
CCD: central composite design
DAO: diamine oxidase
DLS: dynamic light scattering
ATR-FTIR: attenuated total reflectance FTIR
EFSA: European Food Safety Authority
HEK: human embryonic kidney cells
HIT: histamine intolerance
HNMT: histamine *N*-methyltransferase
IONPs: iron oxide nanoparticles
MIP: molecularly imprinted polymer
PBD: Plackett–Burman design
P­(AA-*co*-MAA): poly­(acrylic acid-*co*-methacrylic acid)
PDI: polydispersity index
SDS: sodium dodecyl sulfate
VSM: vibrating sample magnetometry.
