# Supplementary material for: Polymer-Coated Iron Oxide Nanoparticles as an Effective Tool for Histamine Extraction: Synthesis, Characterization, and Application
Source: ACS Omega. 2025 Oct 10;10(51):62839–53. doi: 10.1021/acsomega.5c08198 (PMC12756767; doi:10.1021/acsomega.5c08198)
Supplement: Supplementary file 1 [file ao5c08198_si_001.pdf]

Polymer-coated iron oxide nanoparticles as an effective tool for histamine extraction: Synthesis, characterization, and application

Marco Reindl[1], Anjali Karn[1], Verena Zach[1], Sebastian P. Schwaminger[1,2]*

1 NanoLab, Division of Medicinal Chemistry, Otto Loewi Research Center, Medical University of Graz, Neue Stiftingtalstraße 6, 8010 Graz, Austria

2 BioTechMed-Graz, Mozartgasse 12, 8010 Graz, Austria

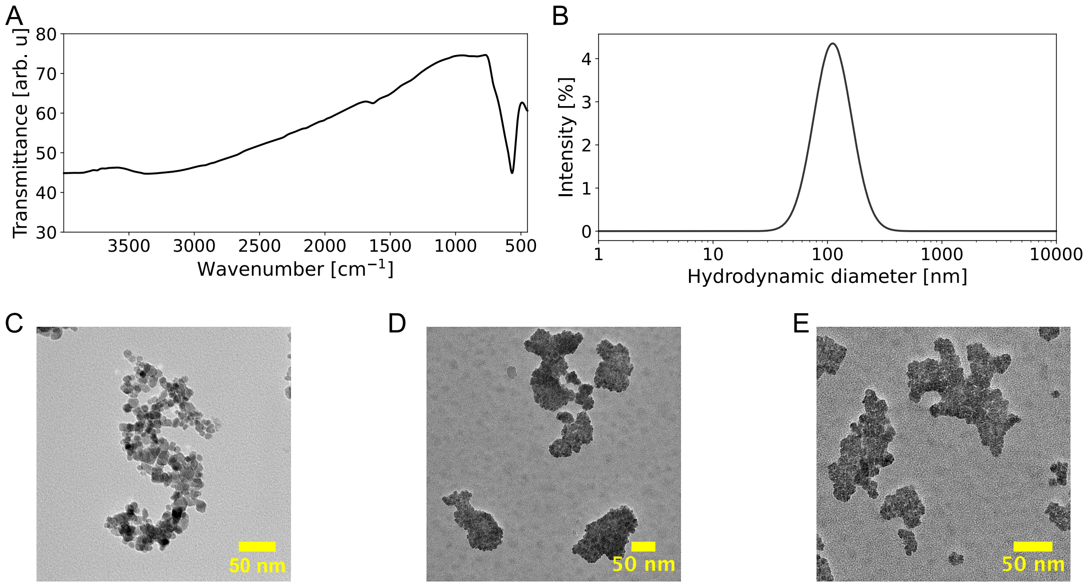

Figure S1. Characterization of bare (BIONs) and polymer-coated IONPs [ION@P(AA-co-MAA)]. (A) FTIR spectra of BIONs. (B) Intensity distribution obtained by DLS. Transmission electron micrographs of (C) BIONs, (D) ION@P(AA-co-MAA) prepared under condition 7 (PBD), and (E) ION@P(AA-co-MAA) prepared under condition 11 (PBD).

Table S1. Physicochemical properties of BIONs, including Z-average hydrodynamic diameter, polydispersity index (PDI), zeta potential, weight loss, and adsorption capacity. DLS and zeta potential measurements were carried out at 25 °C with particle suspensions adjusted to 25 mg/L in ultrapure water at pH 7.2. Adsorption capacity was determined in 25 mM PBS, pH 7.4.

**Z-average diameter [nm]**	92
**PDI**	0.146
**Zeta potential [mV]**	10.3 ± 0.25
**Weight loss [%]**	0.8 ± 0.16
**Adsorption [mg/g]**	11.4 ± 3.45

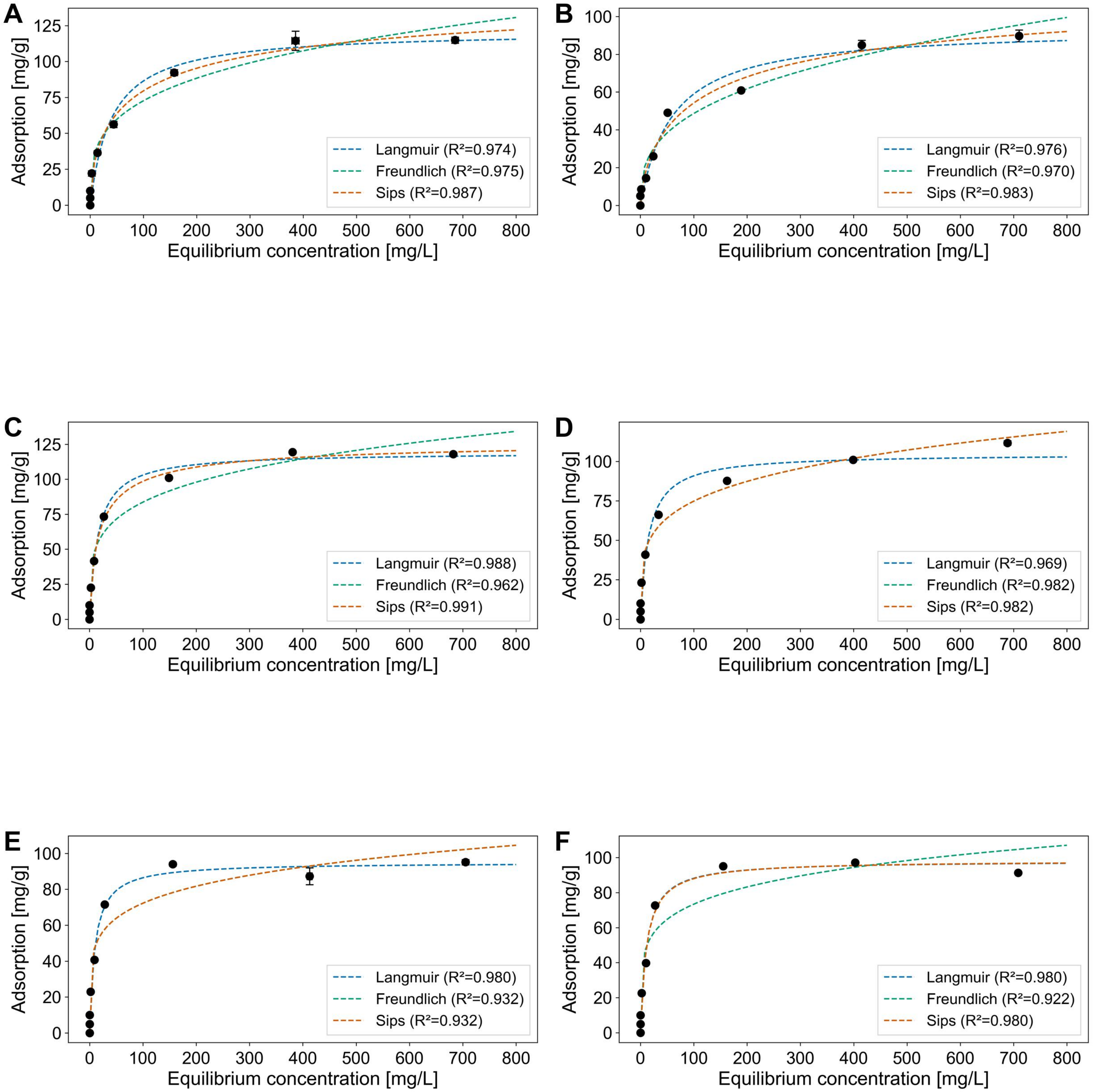
A
Adsorption [mg/g]
Equilibrium concentration [mg/L]
Langmuir (R²=0.974)
Freundlich (R²=0.975)
Sips (R²=0.987)
B
Adsorption [mg/g]
Equilibrium concentration [mg/L]
Langmuir (R²=0.976)
Freundlich (R²=0.970)
Sips (R²=0.983)
C
Adsorption [mg/g]
Equilibrium concentration [mg/L]
Langmuir (R²=0.988)
Freundlich (R²=0.962)
Sips (R²=0.991)
D
Adsorption [mg/g]
Equilibrium concentration [mg/L]
Langmuir (R²=0.969)
Freundlich (R²=0.982)
Sips (R²=0.982)
E
Adsorption [mg/g]
Equilibrium concentration [mg/L]
Langmuir (R²=0.980)
Freundlich (R²=0.932)
Sips (R²=0.932)
F
Adsorption [mg/g]
Equilibrium concentration [mg/L]
Langmuir (R²=0.980)
Freundlich (R²=0.922)
Sips (R²=0.980)

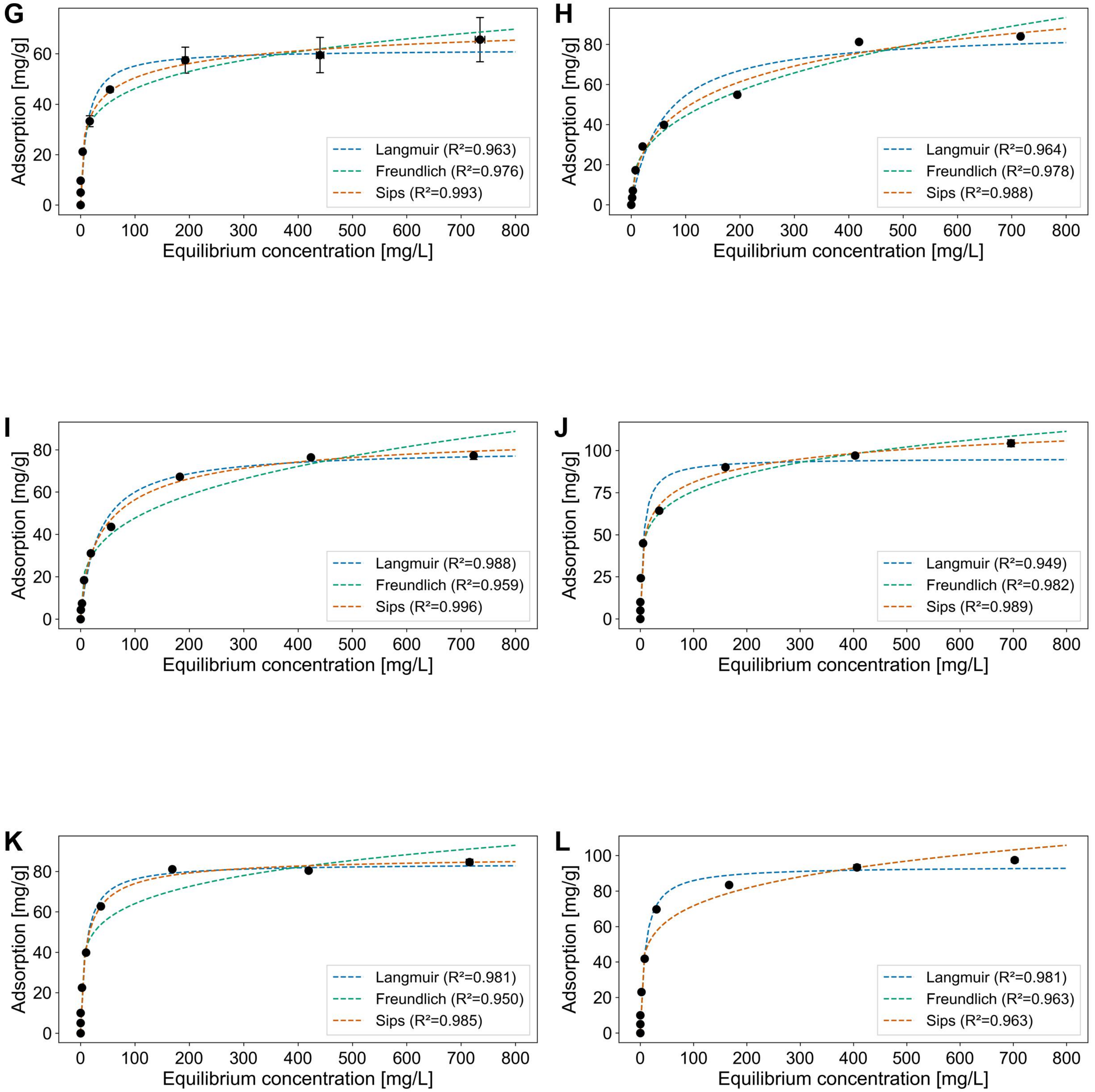
G
Adsorption [mg/g]
Equilibrium concentration [mg/L]
Langmuir (R²=0.963)
Freundlich (R²=0.976)
Sips (R²=0.993)
H
Adsorption [mg/g]
Equilibrium concentration [mg/L]
Langmuir (R²=0.964)
Freundlich (R²=0.978)
Sips (R²=0.988)
I
Adsorption [mg/g]
Equilibrium concentration [mg/L]
Langmuir (R²=0.988)
Freundlich (R²=0.959)
Sips (R²=0.996)
J
Adsorption [mg/g]
Equilibrium concentration [mg/L]
Langmuir (R²=0.949)
Freundlich (R²=0.982)
Sips (R²=0.989)
K
Adsorption [mg/g]
Equilibrium concentration [mg/L]
Langmuir (R²=0.981)
Freundlich (R²=0.950)
Sips (R²=0.985)
L
Adsorption [mg/g]
Equilibrium concentration [mg/L]
Langmuir (R²=0.981)
Freundlich (R²=0.963)
Sips (R²=0.963)

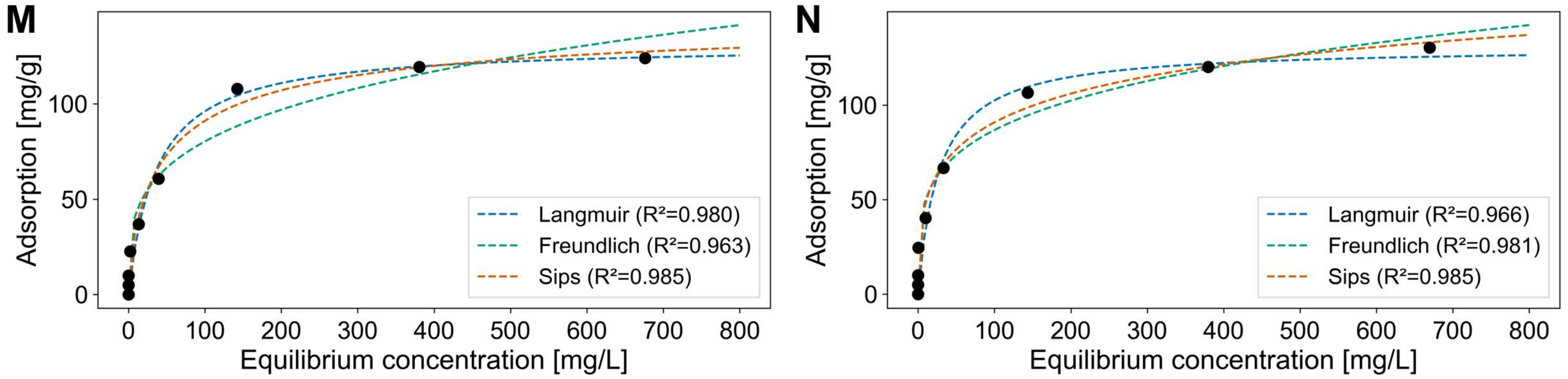

Figure S2. Adsorption isotherms of histamine on ION@P(AA-co-MAA) nanoparticles synthesised under different conditions (D01–D14), (A)–(N). Each isotherm was fitted with Langmuir, Freundlich, and Sips models. Experimental data points represent the mean ± standard deviation of three independent measurements.

Table S2. Statistical evaluation of synthesis factors affecting the hydrodynamic diameter of polymer-coated IONPs using PBD.

**R2**	0.865			
**Adjusted R2**	0.752			
**P(F statistics)**	0.014			
**Factor**	**Coefficient**	**Std. Error**	**t-Statistic**	**p-Value**
Initiator to monomer ratio	-10.1	4.96	-2.043	0.087
Monomer to IONPs ratio	24.3	5.44	4.469	0.004
SDS Concentration	6.00	5.68	1.057	0.331
Polymerisation time	-5.64	5.00	-1.127	0.303
Temperature	-15.8	5.15	-3.066	0.022

Table S3. Influence of synthesis variables on the zeta potential of polymer-coated IONPs, as determined by PBD.

**R2**	0.908			
**Adjusted R2**	0.792			
**P(F statistics)**	0.034			
**Factor**	**Coefficient**	**Std. Error**	**t-Statistic**	**p-Value**
Initiator to monomer ratio	-0.326	0.491	-0.664	0.543
Monomer to IONPs ratio	-1.81	0.587	-3.09	0.037
SDS Concentration	0.611	0.571	1.07	0.345
Polymerisation time	-0.122	0.444	-0.274	0.798
Temperature	-1.48	0.51	-2.91	0.044

Table S4. PBD results evaluating the influence of synthesis factors on the weight loss of polymer-coated IONPs.

**R2**	0.986			
**Adjusted R2**	0.963			
**P(F statistics)**	0.0054			
**Factor**	**Coefficient**	**Std. Error**	**t-Statistic**	**p-Value**
Initiator to monomer ratio	0.193	0.094	2.05	0.133
Monomer to IONPs ratio	0.741	0.114	6.51	0.007
SDS Concentration	-0.375	0.126	-2.99	0.058
Polymerisation time	0.009	0.086	0.11	0.919
Temperature	0.934	0.114	8.21	0.004

Table S5. CCD results assessing the effect of synthesis parameters on the hydrodynamic diameter of polymer-coated iron oxide nanoparticles.

**R2**	0.984			
**Adjusted R2**	0.972			
**P(F statistics)**	0.000004			
**Factor**	**Coefficient**	**Std. Error**	**t-Statistic**	**p-Value**
Monomer to IONPs ratio	200.3	15.52	12.90	0.001
Temperature	-17.00	12.99	-1.31	0.232
Monomer to IONPs ratio**2	347.8	28.20	12.3	0.001
Temperature**2	-14.75	27.191	-0.542	0.604
Monomer to IONPs ratio : Temperature	-26.3283	15.527	-1.696	0.134

Table S6. Evaluation of synthesis parameter effects on the zeta potential of polymer-coated IONPs using a CCD approach.

**R2**	0.997			
**Adjusted R2**	0.995			
**P(F statistics)**	0.001			
**Factor**	**Coefficient**	**Std. Error**	**t-Statistic**	**p-Value**
Monomer to IONPs ratio	-6.34	0.134	-47.50	0.001
Temperature	0.006	0.120	0.054	0.958
Monomer to IONPs ratio**2	0.023	0.208	0.133	0.897
Temperature**2	0.210	0.209	1.00	0.344
Monomer to IONPs ratio : Temperature	-0.13	0.143	-0.905	0.392

Table S7. Assessment of the impact of synthesis parameters on the weight loss of polymer-coated IONPs using CCD.

**R2**	0.911			
**Adjusted R2**	0.847			
**P(F statistics)**	0.0015			
**Factor**	**Coefficient**	**Std. Error**	**t-Statistic**	**p-Value**
Monomer to IONPs ratio	0.579	0.029	20.2	0.001
Temperature	0.682	0.026	26.5	0.001
Monomer to IONPs ratio**2	-0.020	0.045	-0.444	0.669
Temperature**2	0.002	0.045	0.043	0.967
Monomer to IONPs ratio : Temperature	0.127	0.031	4.12	0.019

Table S8. CCD analysis of factors affecting histamine adsorption and retention on polymer-coated iron oxide nanoparticles.

**R2**	0.902			
**Adjusted R2**	0.82			
**P(F statistics)**	0.0055			
**Factor**	**Coefficient**	**Std. Error**	**t-Statistic**	**p-Value**
Monomer to IONPs ratio	-17.02	8.04	-2.12	0.078
Temperature	6.4	6.08	1.05	0.333
Monomer to IONPs ratio**2	-25.95	10.52	-2.47	0.049
Temperature**2	-26.22	7.01	-3.74	0.010
Monomer to IONPs ratio : Temperature	11.17	8.04	1.39	0.214

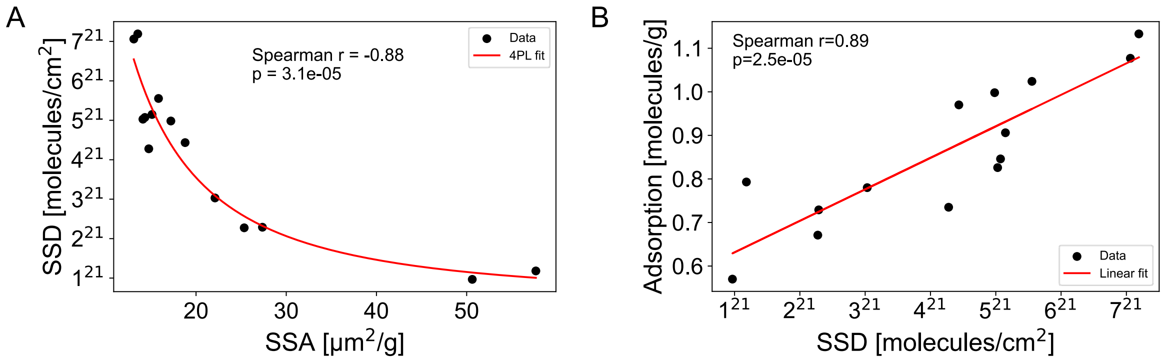

Figure S3. Analysis of histamine adsorption data showing the relationship between (A) specific site density (SSD) and adsorption capacity using a linear regression, and between (B) specific surface area (SSA) and SSD using a four-parameter logistic regression (4PL). Statistical significance was evaluated using Spearman correlation.

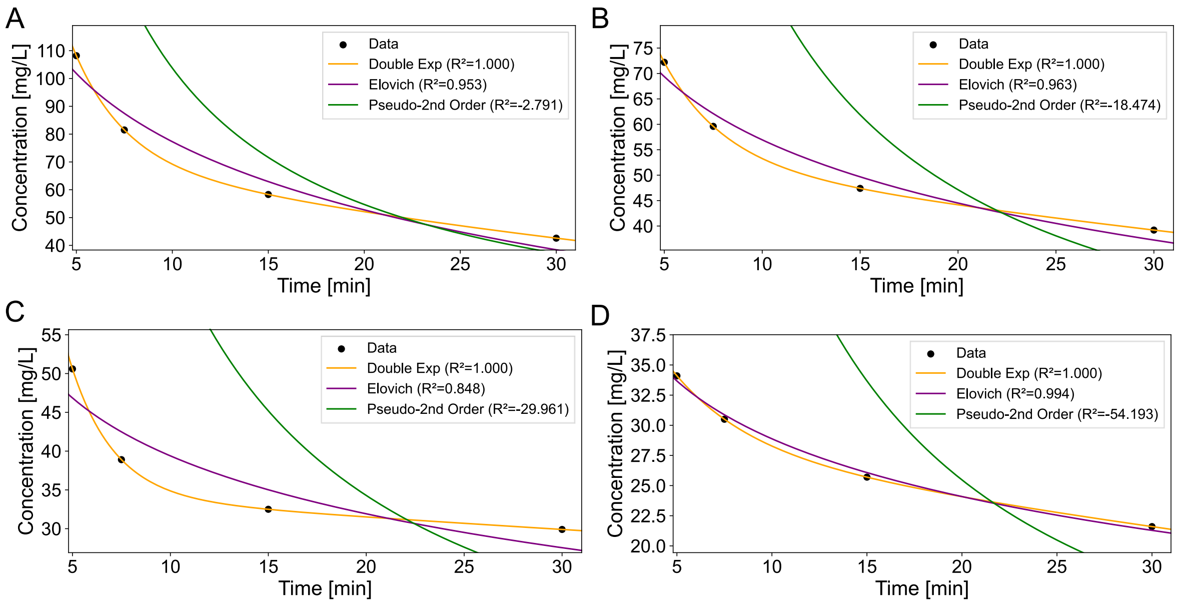

Figure S4. Separation kinetics in beer-mimicking buffer by centrifugation with the indicated fit and the respective R2 value at (a), 5,000 x g, (b) 10,000 x g, (c) 15,000 x g, and (d) 20,000 x g.

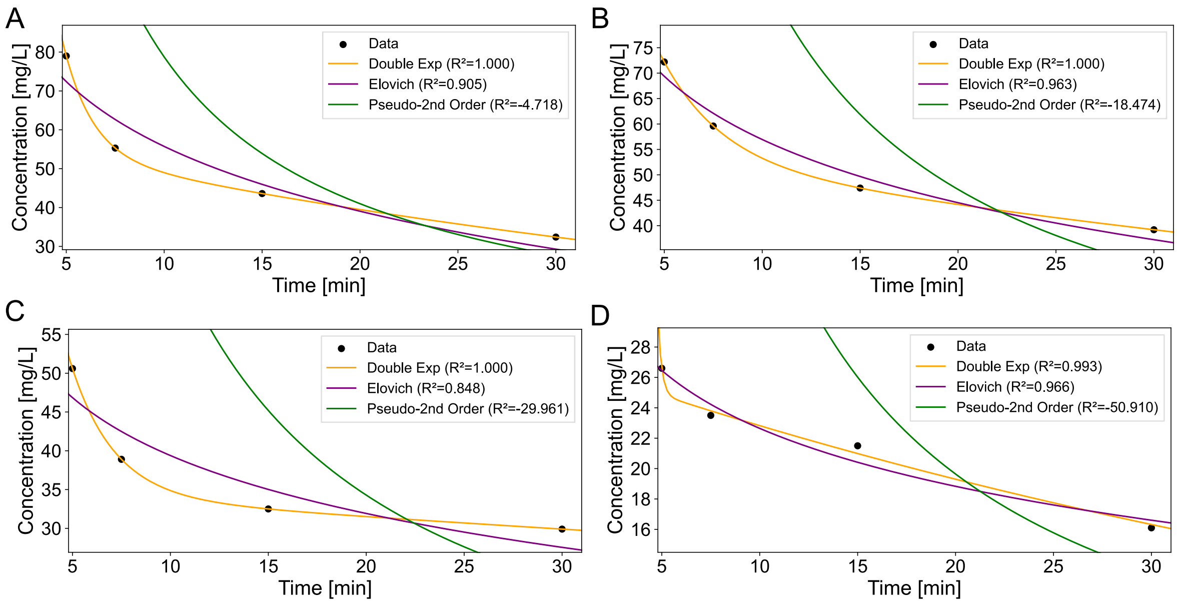

Figure S5. Separation kinetics in soy sauce-mimicking buffer by centrifugation with the indicated fit and the respective R2 value at (a), 5,000 x g (b) 10,000 x g, (c) 15,000 x g, (d) 20,000 x g.

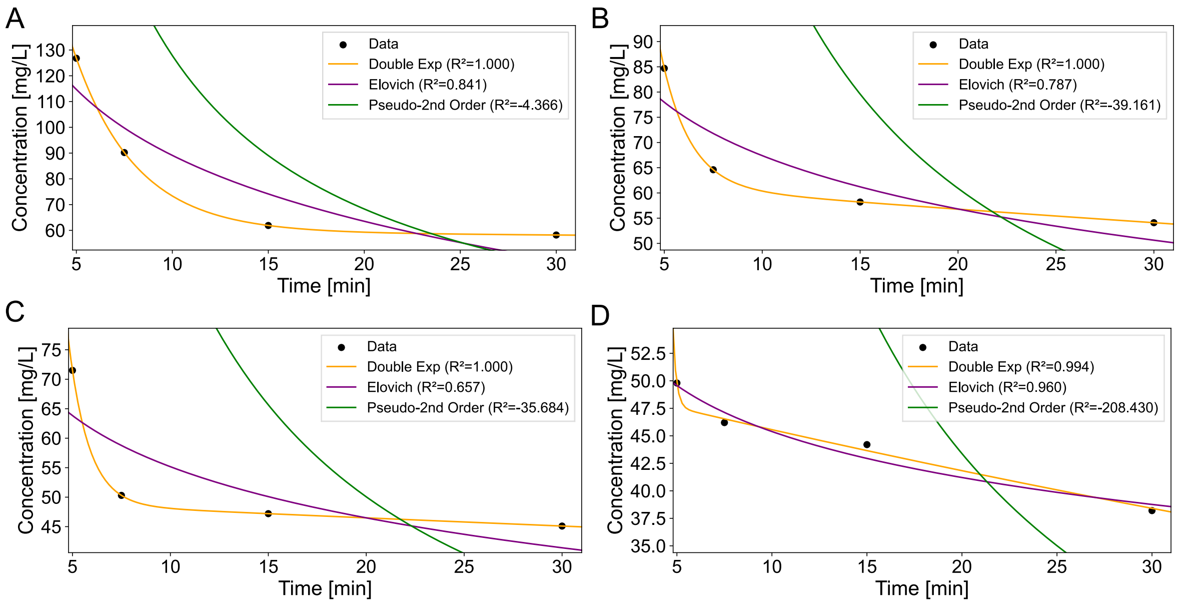

Figure S6. Separation kinetics in wine-mimicking buffer by centrifugation with the indicated fit and the respective R2 value at (a), 5,000 x g (b) 10,000 x g, (c) 15,000 x g, (d) 20,000 x g.
